# Amputation

**Published:** 1855-03

**Authors:** 


ART. II.—*Amputation.*

[This paper was read before the La Salle County Medical Society, on the 6th of December, 1854, and requested to be published in the Chicago North Western Medical Journal, by said Society.] THEODORE HAY, Pres't.
J. O. HARRIS, Sec.

On the 21st of July, 1854, at 2 o'clock P. M.. Mr. A. Onderkirk was run over by a mail train on the C. & R. I. R. R., crushing the lower third of his left leg, so as to render amputation necessary. Owing to the kindly influence of gentlemen having business connection with said road, Dr. ——, was called to attend the case. But he not having necessary instruments to perform the operation, called to his aid one of the oldest surgeons of the city of Peru; thereby securing not only the necessary instruments, but also the skill of the old surgeon. The two surgeons met at 2½ o'clock, P.M., and proceeded at once to administer chloroform till the patient was profoundly insensible to all pain; in which situation (with praiseworthy humanity) they kept him till the operation was completed, which was just three hours.

The circular operation was performed as follows: The patient was placed upon an ordinary dining table, the operator being seated on a chair at his left side, with all the instruments of the two displayed on a table in his rear at his left. The next step was to encircle the limb with a thread, to serve as a guide to the operator in his first incision, which divided the skin cellular tissue, and about one half of the gastronemius muscle. Next the skin and cellular tissue were dissected back about two inches with a concave edged circular knife.

The second incission was made to correspond with the first, and the bone sawed off at the same point; that is to say, the limb was cut off as a butcher would cut a steak from the round of the thigh of an ox; the skin, muscles, and bone all on the same plane. The arteries were secured in the usual manner, and an attempt made to cover the stump with the integuments; but as *some* might have anticipated, they were found too short. Nothing daunted, however, the operator re-applied the saw, which left the stump of tibia minus another inch and a half in length, and the fibula (of course) one and half inches longer than it (the tibia.) Now the hair was shaved from the amputated limb, and another attempt made to cover it with the integuments. But, lo! notwithstanding much force was used, by means of four interrupted sutures, still the stubborn integuments refused to cover the stump, but left a portion of the gastronemius muscle protruding about the size of a hen's egg. But science is full of expedients, and this refractory portion of muscle soon fell a victim to the surgeon's knife; and all appearances indicated that science had completely triumphed over nature; when a vicious little artery spurted into the face and eyes of the surgeon, a sure indication that his work was not yet done. This little rebel was, however, soon subdued, and nothing now (five months after the accident) remains to mar the beauty and benefit of this important operation, except a small portion of the stump of this amputated limb (say about two and a half inches in circumference) remains unhealed, with the end of the fibula protruding from its centre. JOHN HIGGINS, M.D.

Peru, Dec. 6th 1854.

